# Supplementary material for: The interactome of intact mitochondria by cross-linking mass spectrometry provides evidence for coexisting respiratory supercomplexes
Source: Mol Cell Proteomics. 2017 Dec 8;17(2):216–32. doi: 10.1074/mcp.RA117.000470 (PMC5795388; doi:10.1074/mcp.RA117.000470)
Supplement: Supplemental Data [file supp_RA117.000470_133922_0_supp_23978_fzffwf.zip › spectra_annotation/mito_DR_spectra_annotation/300-1-3-1-4-1.pdf]

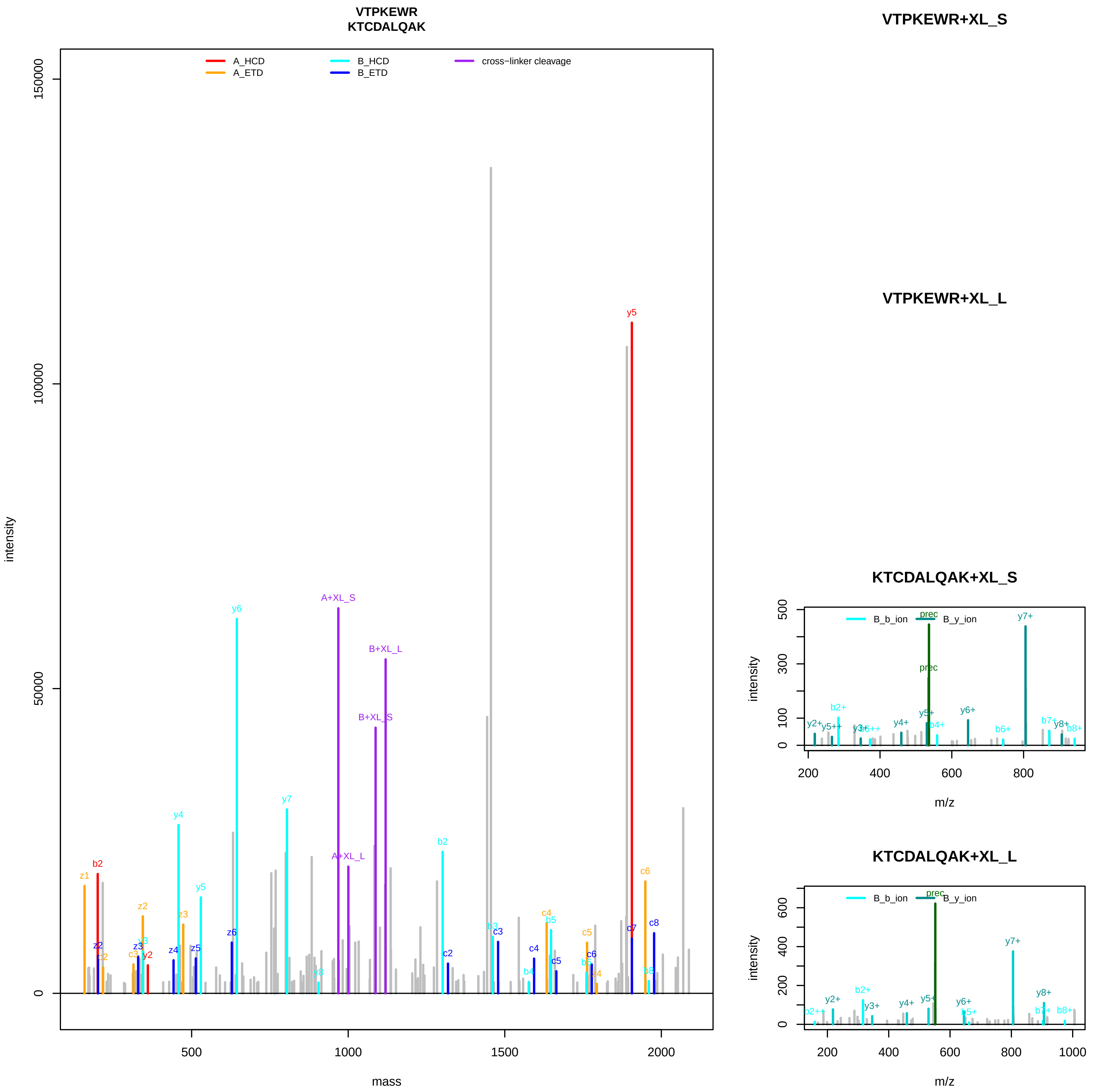

VTPKEWR
KTCDALQAK
A_HCD
A_ETD
B_HCD
B_ETD
cross−linker cleavage
intensity
mass
0
50000
100000
150000
500
1000
1500
2000
y5
A+XL_S
B+XL_L
B+XL_S
A+XL_L
y6
y7
y4
y5
b2
z1
z2
z3
c2
c3
c4
c5
c6
z4
y2
y3
z2
z3
z4
z5
z6
c2
c3
c4
c5
c6
c7
c8
b3
b4
b5
b6
b8
y8
VTPKEWR+XL_S
VTPKEWR+XL_L
KTCDALQAK+XL_S
B_b_ion
B_y_ion
prec
y7+
y2+
y5++
b2+
y3+
b6++
y4+
y5+
b4+
y6+
b6+
b7+
y8+
b8+
intensity
m/z
0
100
300
500
200
400
600
800
KTCDALQAK+XL_L
B_b_ion
B_y_ion
prec
y7+
b2++
y2+
b2+
y3+
y4+
y5+
y6+
b5+
y8+
b7+
b8+
intensity
m/z
0
200
400
600
200
400
600
800
1000
